# Estimation of the stress-strength reliability for the inverse Weibull distribution under adaptive type-II progressive hybrid censoring

**DOI:** 10.1371/journal.pone.0277514

**Published:** 2022-11-15

**Authors:** Majd Alslman, Amal Helu

**Affiliations:** Department of Mathematics, The University of Jordan, Amman, Jordan; Georgia Southern University, UNITED STATES

## Abstract

In this article, we compare the maximum likelihood estimate (MLE) and the maximum product of spacing estimate (MPSE) of a stress-strength reliability model, *θ* = *P*(*Y* < *X*), under adaptive progressive type-II progressive hybrid censoring, when X and Y are independent random variables taken from the inverse Weibull distribution (IWD) with the same shape parameter and different scale parameters. The performance of both estimators is compared, through a comprehensive computer simulation based on two criteria, namely bias and mean squared error (MSE). To demonstrate the effectiveness of our proposed methods, we used two examples of real-life data based on Breakdown Times of an Insulated Fluid by (Nelson, 2003) and Head and Neck Cancer Data by (Efron, 1988). It is concluded that the MPSE method outperformed the MLE method in terms of bias and MSE values.

## Introduction

In many life-testing experiments, the experimenter faces different challenges to control the test time and to conserve experimental units while estimating efficiently and this can be achieved by stopping the experiment before all units fail by using censoring schemes that are carried out by removing active units from the experiment.

During these experiments, units may be lost or removed for different reasons, and this is where the importance of progressive censoring arises in which units are removed under a life test experiment at some predetermined or random time points during the experiment.

Many models of progressive censoring have been discussed throughout the years. The majority of these models can be traced back to one of two sources: progressive type-I censoring, which terminates the experiment after a prefixed time point, or progressive type-II censoring, which terminates the experiment after a prefixed number of observed failures. Both censoring schemes give the experimenter more flexibility by allowing the removal of test units at non-terminal time points during the experiment.

In progressive type-I censoring, the total time of the experiment is predetermined, and the censoring occurs at prefixed time points *T*_1_, *T*_2_, …, *T*_*r*_. A prefixed number of active units are removed during the experiment at the end of each specified time intervals, making the number of observed failure lifetimes random. Hence, in type-I progressive censoring one might observe a few, if any, failures when units under the test have long lifetimes.

In progressive type-II censoring scheme, only m units are completely observed until failure, out of n units placed on a life-test. When the first failure occurs, *R*_1_ active units are removed from the *n* − 1 remaining units. After the second failure, *R*_2_ active units are removed from the *n* − *R*_1_ − 2 remaining units. Lastly, at the m-th failure, all the remaining *n* − *R*_1_ − *R*_2_…−*R*_*m*−1_ units are removed and the experiment is terminated. since the time of the experiment is random, when units undertaking the life test have long life times it results in a long test duration, which is considered a disadvantage for progressive type-II censoring.

Two progressive hybrid censoring schemes were proposed by [1] by stopping a progressive type-II censoring experiment at time T*. In type-I progressive hybrid censoring scheme, T* = *min*(*X*_*m*:*n*_, *T*)such that, *X*_*m*:*n*_ is the time of the m-th failure and *T* is a stopping time that is predetermined by the experimenter. In this case, we may have fewer than m observations when units undergoing the test have long failure times. In type-II progressive hybrid censoring scheme, T* = *max*(*X*_*m*:*n*_, *T*), we may have at least m observations but a long test duration.

**Fig 1:**
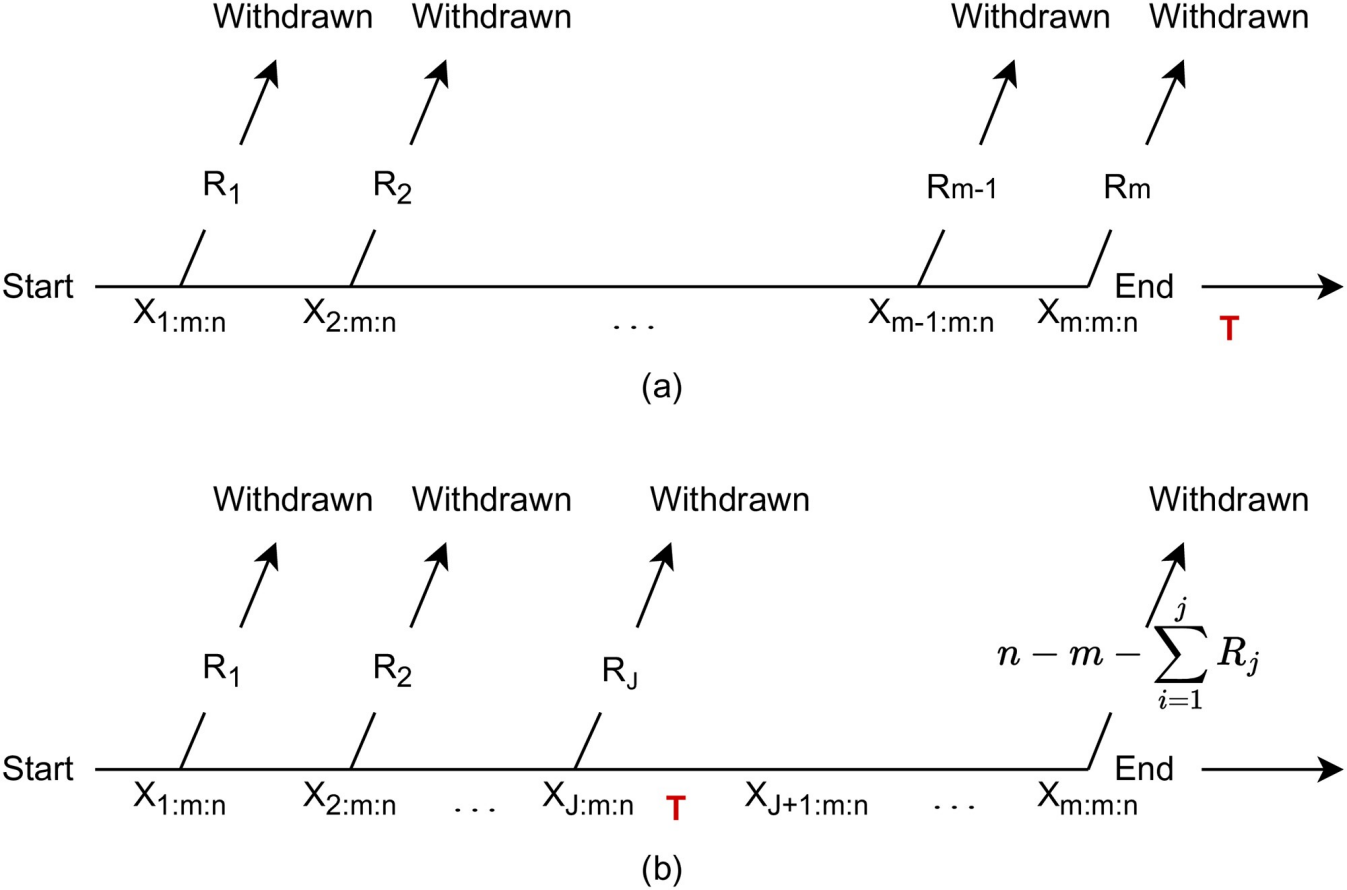
Adaptive type-II progressive hybrid censoring model as proposed by Ng et al., (2009). (a) Experiment ends before time T. (b) Experiment ends after time T.

In real-life experiments, having a fixed censoring scheme may not be convenient because the censoring scheme may change, intentionally or unintentionally, during the experiment. [2] proposed a newer model (see Fig 1), which allows changing the censoring scheme during the experiment. Such model is called the adaptive type-II progressive hybrid censoring, in which a threshold time T is used to switch from the initially planned censoring scheme to a modified one.

In a sample of size n, m failures will be observed, after a threshold time T, the censoring number *R*_*j*_, *j* = *max*(*i*, *X*_*i*: *m*: *n*_ < *T*), will adaptively change based on the previous failure times as well as the censored samples before the j-th failure. That is, after the first observed failure time that exceeds the threshold time T, the applied censoring scheme will be changed to $R^{*}=\left(R_{1},\ldots ,R_{j},0,\ldots ,0,n-m-\sum_{i=1}^{j}R_{j}\right)$. The initially planned progressive censoring scheme is used as long as the failures occur before time T (see Fig 1(a)). Otherwise, when time T occurs before the m-th failure, no units are withdrawn after time T except for the time of the m-th failure where all remaining surviving units are removed (see Fig 1(b)). By setting *T* = ∞ and *T* = 0, we get type-II progressive censoring and type-II censoring, respectively.

Failure times of units under a life-testing experiment are assumed to be identically distributed and follow a lifetime distribution. One of the most widely used lifetime distributions to model progressive censoring schemes is the Weibull distribution (WD), named after the Swedish mathematician Waloddi Weibull.

If a random variable T follows the WD with a shape parameter *α* and a scale parameter *β*, then the probability density function (PDF) is given by Eq 1

$$\begin{array}{c} f\left(t;\alpha ,\beta \right)=\alpha \beta e^{-\beta t^{\alpha }}t^{-1+\alpha }\;t>0,\alpha ,\beta >0 \end{array}$$ (1)

and the hazard function (HF), which measures the probability of failure of a unit at a given time, is given by Eq 2

$$\begin{array}{c} h\left(t;\alpha ,\beta \right)=\alpha \beta^{-\alpha }t^{-1+\alpha }\;t>0 \end{array}$$ (2)

**Fig 2:**
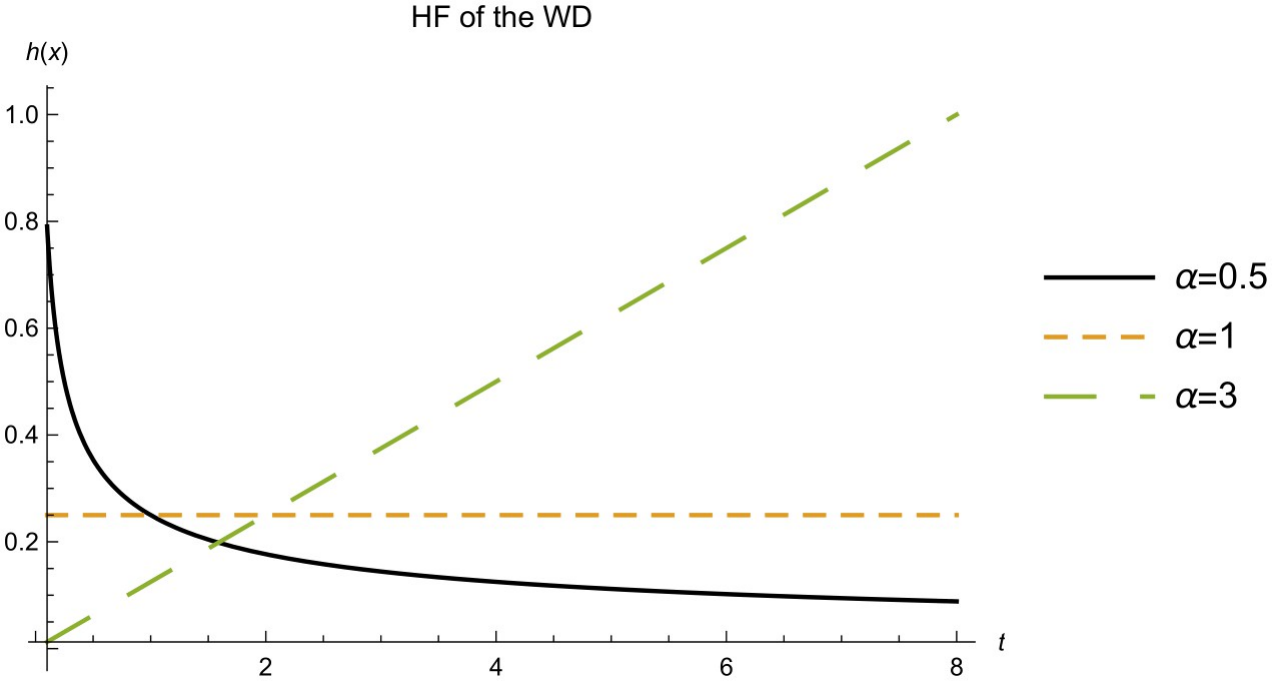
HF of the WD. The HF of the WD with scale parameter *β* = 4 and different values for the shape parameter *α*.

The HF of the WD given in Eq 2 can not be used to model life time data with a bath tub shaped hazard function, since it is increasing, decreasing, or constant as shown in Fig 2. This is considered a drawback for the WD.

The Inverse Weibull distribution (IWD), also known as the type-II extreme value distribution or the Frechet distribution [3], is used to model a variety of failure characteristics such as infant mortality (early failure), useful life, and wear-out periods (the increase of the number of failure occurrences after a certain usage period) [4].

**Fig 3:**
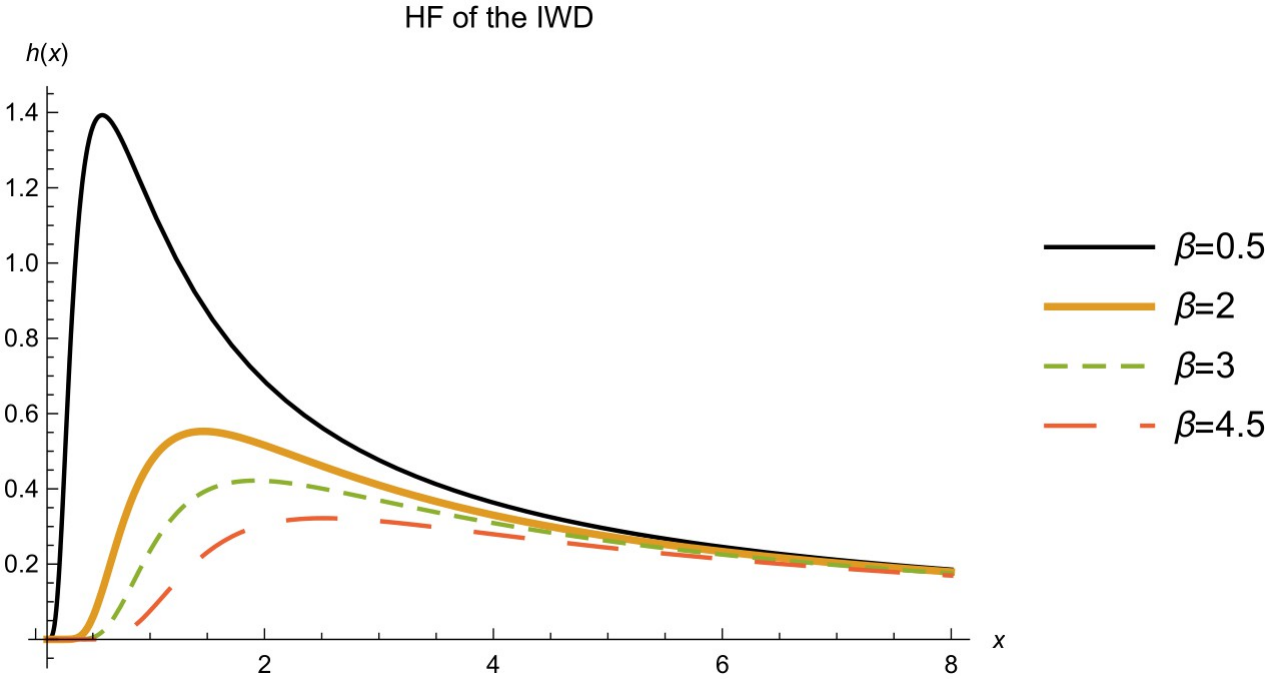
HF of the IWD. HF of the IWD with a shape parameter *α* = 1.5 and different values for the scale parameter *β*.

The HF of the IWD given in Eq 4, is uni-modal, see Fig 3. Having a uni-modal hazard function is essential in many practical situations where the risk increases and then decreases as the study continues, like the process of recovery after a patient undergoes a surgery.

If $X=\frac{1}{T}$, then *X* follows the IWD with PDF, cumulative distribution function (CDF), and HF given by Eqs 3, 4 and 5 respectively.

$$\begin{array}{c} f\left(x;\alpha ,\beta \right)=\alpha \beta e^{-\beta x^{-\alpha }}x^{-1-\alpha }\;x>0,\alpha ,\beta >0, \end{array}$$ (3)

$$\begin{array}{c} F\left(x;\alpha ,\beta \right)=e^{-\beta x^{-\alpha }}\;x>0, \end{array}$$ (4)

$$\begin{array}{c} h\left(x;\alpha ,\beta \right)=\frac{\alpha \beta x^{-\alpha -1}}{e^{\beta x^{-\alpha }}-1}. \end{array}$$ (5)

**Fig 4:**
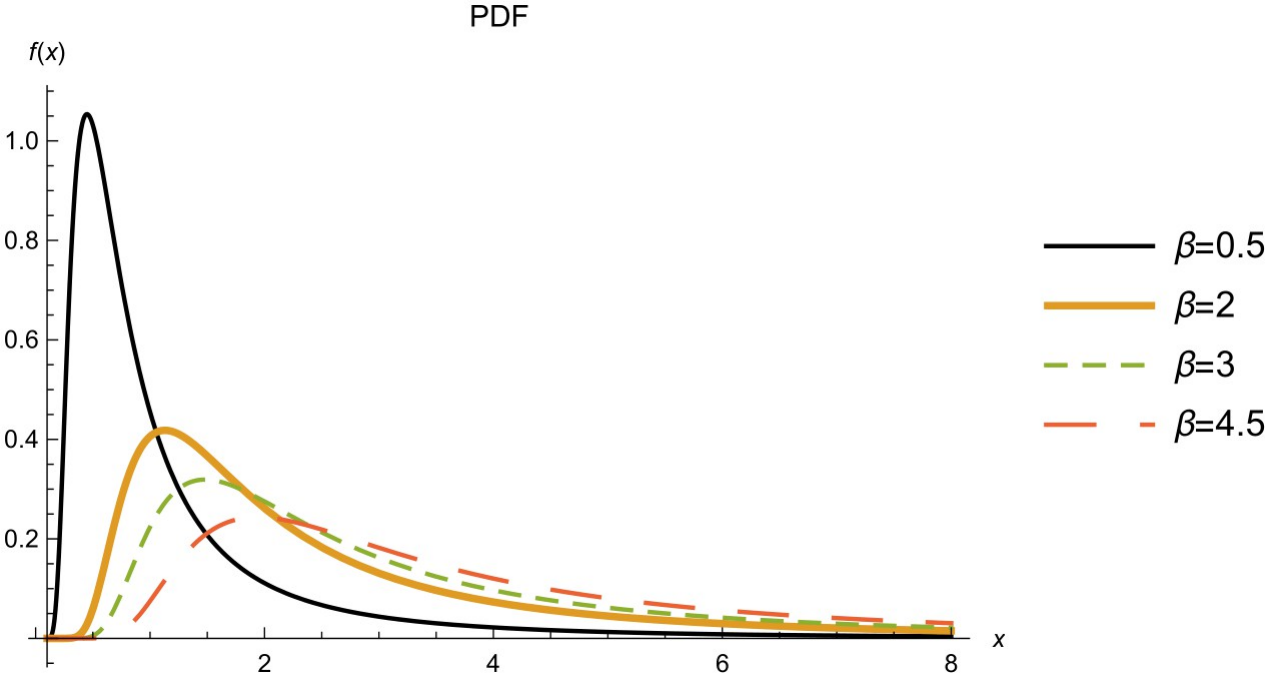
PDF of the IWD when. *α* = 1.5.

**Fig 5:**
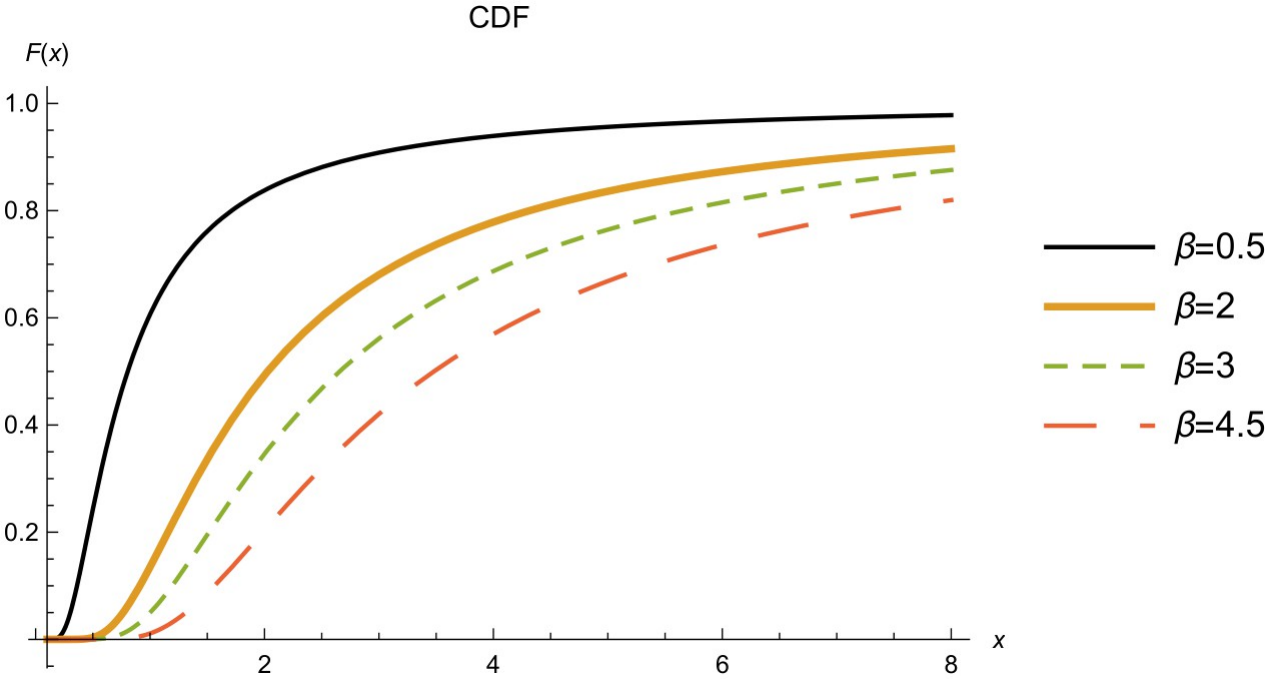
CDF of the IWD when. *α* = 1.5.

Figs 4 and 5 show the PDF and CDF of the IWD for different scale parameter values *β*.

Many studies have considered the IWD under progressive censoring, see for example [5] estimated the unknown parameters of the three-parameter IWD and as a result obtained a theorem on the existence of the least squares estimates, [6] considered statistical inferences about the unknown parameters of the IWD based on progressively type-II censoring using the maximum likelihood, least squares estimators, and the approximate maximum likelihood estimators as well as the Bayes estimators using Lindley’s approximation method and symmetric and asymmetric loss functions, and for recent references, see [7–9].

The estimation of a stress-strength reliability (SSR) model *P*(*Y* < *X*) was first introduced by [10], who used properties of the Mann and Whitney statistic to estimate *P*(*Y* < *X*) where X and Y are random variables with continuous cumulative probability functions. The Mann and Whitney Statistic is based on the ranks of observations on X and Y in a joint sample.

In this model, a failure will occur when a component with relatively low strength X is paired off with a high-stress Y. The stress Y could be mechanical, temperature, or voltage stress, while the strength X could be any resisting physical property like hardness, melting point, or adhesion. *P*(*Y* < *X*) is then used to measure the probability of the system not failing under the applied stress. *P*(*Y* < *X*) can also be used to measure the probability of a random sample Y having shorter survival times than a random sample X. Many applications of this model can be found in [11].

Statistical inference of the SSR model under complete sample case and progressive censoring using classical and Bayesian approximation methods was studied by many authors such as: [12–17].

The main interest of this study is to compare different methods of estimation of a stress-strength model *θ* = *P*(*Y* < *X*), where X and Y are two independent IWD variables under adaptive type-II hybrid progressive censoring with the same shape parameter and different scale parameters. Where X represents the strength of a unit and Y represents the stress applied to the unit.

## Estimation methods

### Maximum likelihood estimation

Suppose *X* ∼ *IWD*(*α*, *β*_1_) and *Y* ∼ *IWD*(*α*, *β*_2_) are two independent random variables representing the strength and stress components, respectively. Then, the SSR model is given by Eq 6.

$$\begin{array}{c} \begin{array}{cc} \theta =P(Y<X) & =\int_{0}^{\infty }f\left(x|\alpha ,\beta_{1}\right)P\left(Y\leq x\right)dx\\ & =\int_{0}^{\infty }f\left(x|\alpha ,\beta_{1}\right)F\left(x|\alpha ,\beta_{2}\right)dx\\ & =\int_{0}^{\infty }\alpha \beta_{1}x^{-(1+\alpha )}e^{-\left(\beta_{1}+\beta_{2}\right)x^{-\alpha }}dx\\ & =\frac{\beta_{1}}{\beta_{1}+\beta_{2}} \end{array} \end{array}$$ (6)

Since *θ* is a function of *β*_1_ and *β*_2_, one can obtain the MLE of *θ* (*θ*_*MLE*_) using the invariance property of the MLE by calculating the MLE of *β*_1_ and *β*_2_ $\left(\beta_{1_{MLE}},\beta_{2_{MLE}}\right)$.

Let $X=(X_{1:m_{1}:n_{1}},X_{2:m_{1}:n_{1}},\ldots ,X_{m_{1}:m_{1}:n_{1}})$ with $\left\{X_{1:m_{1}:n_{1}}<X_{2:m_{1}:n_{1}}<\ldots <X_{m_{1}:m_{1}:n_{1}}\right\}$ be an adaptive type-II hybrid progressive censoring sample from *IW*(*α*, *β*_1_) under the censoring scheme $\left\{n_{1},m_{1},R_{1},\ldots ,R_{J_{1}},0,\ldots ,0,R_{m_{1}}=n_{1}-m_{1}-\sum_{i=1}^{J_{1}}R_{i}\right\}$ such that $X_{J_{1}:m_{1}:n_{1}}<T_{1}<X_{J_{1}+1:m_{1}:n_{1}}$.

Similarly, let $Y=(Y_{1:m_{2}:n_{2}},Y_{2:m_{2}:n_{2}},\ldots ,Y_{m_{2}:m_{2}:n_{2}})$ with $\left\{Y_{1:m_{2}:n_{2}}<Y_{2:m_{2}:n_{2}}<\ldots <Y_{m_{2}:m_{2}:n_{2}}\right\}$ be an adaptive type-II hybrid progressive censoring sample from *IW*(*α*, *β*_2_) under the scheme $\left\{n_{2},m_{2},S_{1},\ldots ,S_{J_{2}},0,\ldots ,0,S_{k}=n_{2}-m_{2}-\sum_{i=1}^{J_{2}}S_{i}\right\}$ such that $Y_{J_{2:m_{2}:n_{2}}}<T_{2}<Y_{J_{2}+1:m_{2}:n_{2}}$.

For simplicity, let $X_{i}=X_{i:m_{1}:n_{1}}$ and $Y_{i}=Y_{i:m_{2}:n_{2}}$. The joint likelihood function of the adaptive type-II hybrid progressively censored sample (see [18]) can be written as shown in Eq 7.

$$\begin{array}{c} \begin{array}{cc} L(\alpha ,\beta_{1},\beta_{2}|X,Y) & =C_{1}C_{2}{\left[1-F_{1}\left(x_{m_{1}}\right)\right]}^{R_{m_{1}}}\prod_{i=1}^{m_{1}}f_{1}\left(x_{i}\right)\prod_{i=1}^{J_{1}}{\left[1-F_{1}\left(x_{i}\right)\right]}^{R_{i}}\\ & {\left[1-F_{2}\left(y_{m_{2}}\right)\right]}^{S_{m_{2}}}\prod_{i=1}^{m_{2}}f_{2}\left(y_{i}\right)\prod_{i=1}^{J_{2}}{\left[1-F_{2}\left(y_{i}\right)\right]}^{S_{i}} \end{array} \end{array}$$ (7)

Where,

$$\begin{array}{c} C_{1}=n_{1}\left(n_{1}-R_{1}-1\right)\left(n_{1}-R_{1}-R_{2}-2\right)\ldots \left(n_{1}-R_{1}-R_{2}-\ldots -R_{m_{1}-1}-m_{1}+1\right) \end{array}$$

$$\begin{array}{c} C_{2}=n_{2}\left(n_{2}-S_{1}-1\right)\left(n_{2}-S_{1}-S_{2}-2\right)\ldots \left(n_{2}-S_{1}-S_{2}-\ldots -S_{m_{2}-1}-m_{2}+1\right) \end{array}$$

$$\begin{array}{c} f_{1}\left(x;\alpha ,\beta_{1}\right)=\alpha \beta_{1}e^{-\beta_{1}x^{-\alpha }}x^{-1-\alpha }\;x>0,\alpha ,\beta_{1}>0. \end{array}$$

$$\begin{array}{c} f_{2}\left(y;\alpha ,\beta_{2}\right)=\alpha \beta_{2}e^{-\beta_{2}y^{-\alpha }}y^{-1-\alpha }\;y>0,\alpha ,\beta_{2}>0 \end{array}$$

$$\begin{array}{c} F_{1}\left(x;\alpha ,\beta_{1}\right)=e^{-\beta_{1}x^{-\alpha }}\;x>0 \end{array}$$

$$\begin{array}{c} F_{2}\left(y;\alpha ,\beta_{2}\right)=e^{-\beta_{2}y^{-\alpha }}\;y>0 \end{array}$$

After simplifying Eq 9, the likelihood function can be written as shown in Eq 8.

$$\begin{array}{c} \begin{array}{cc} L(\alpha ,\beta_{1},\beta_{2}|X,Y) & =C_{1}\alpha^{m_{1}+m_{2}}\beta_{1}^{m_{1}}{\left(1-e^{-\beta_{1}x_{m_{1}}^{-\alpha }}\right)}^{R_{m_{1}}}\prod_{i=1}^{m_{1}}\left(e^{-\beta_{1}x_{i}^{-\alpha }x_{i}^{-1-\alpha }}\right)\prod_{i=1}^{J_{1}}{\left(1-e^{-\beta_{1}x_{i}^{-\alpha }}\right)}^{R_{i}}\\ & C_{2}\beta_{2}^{m_{2}}{\left(1-e^{-\beta_{2}y_{m_{2}}^{-\alpha }}\right)}^{S_{m_{2}}}\prod_{i=1}^{m_{2}}\left(e^{-\beta_{2}y_{i}^{-\alpha }y_{i}^{-1-\alpha }}\right)\prod_{i=1}^{J_{2}}{\left(1-e^{-\beta_{2}y_{i}^{-\alpha }}\right)}^{S_{i}} \end{array} \end{array}$$ (8)

The log-likelihood function, based on Eq 8 is given in Eq 9.

$$\begin{array}{c} \begin{array}{cc} l(\alpha ,\beta_{1},\beta_{2}|X,Y) & =\text{ln}\;C_{1}+\text{ln}\;C_{2}+\left(m_{1}+m_{2}\right)\text{ln}\;\alpha +m_{1}\;\text{ln}\;\beta_{1}+m_{2}\;\text{ln}\;\beta_{2}\\ & +R_{m_{1}}\;\text{ln}\;\left(1-e^{-\beta_{1}x_{m_{1}}^{-\alpha }}\right)-\left(1+\alpha \right)\sum_{i=1}^{m_{1}}ln\left(x_{i}\right)-\beta_{1}\sum_{i=1}^{m_{1}}x_{i}^{-\alpha }\\ & +\sum_{i=1}^{J_{1}}R_{i}\;\text{ln}\left(1-e^{-\beta_{1}x_{i}^{-\alpha }}\right)+S_{m_{2}}\;\text{ln}\left(1-e^{-\beta_{2}y_{m_{2}}^{-\alpha }}\right)\\ & -\left(1+\alpha \right)\sum_{i=1}^{m_{2}}\;\text{ln}\left(Y_{i}\right)-\beta_{2}\sum_{i=1}^{m_{2}}y_{i}^{-\alpha }+\sum_{i=1}^{J_{2}}s_{i}\;\text{ln}\left(1-e^{-\beta_{2}y_{i}^{-\alpha }}\right) \end{array} \end{array}$$ (9)

If the shape parameter *α* is known, the maximum likelihood estimators ${\tilde{\beta }}_{1}$ and ${\tilde{\beta }}_{2}$ are derived by maximizing the log-likelihood function, it is computationally easier to maximize by deriving Eq 9 with respect to *β*_1_ and *β*_2_ and equating both resulted equations to zero.

$$\begin{array}{c} \frac{\partial l}{\partial \beta_{1}}=\frac{m_{1}}{\beta_{1}}+\frac{e^{-\beta_{1}x_{m_{1}}^{-\alpha }}R_{m_{1}}x_{m_{1}}^{-\alpha }}{1-e^{-\beta_{1}x_{m_{1}}^{-\alpha }}}-\sum_{i=1}^{m_{1}}x_{i}^{-\alpha }+\sum_{i=1}^{J_{1}}\frac{e^{-\beta 1x_{i}^{-\alpha }}R_{i}x_{i}^{-\alpha }}{1-e^{-\beta_{1}x_{i}^{-\alpha }}}=0 \end{array}$$ (10)

$$\begin{array}{c} \frac{\partial l}{\partial \beta_{2}}=\frac{m_{2}}{\beta_{2}}+\frac{e^{-\beta_{2}y_{m_{2}}^{-\alpha }}S_{m_{2}}y_{m_{2}}^{-\alpha }}{1-e^{-\beta_{2}y_{m_{2}}^{-\alpha }}}-\sum_{i=1}^{m_{2}}y_{i}^{-\alpha }+\sum_{i=1}^{J_{2}}\frac{e^{-\beta 2y_{i}^{-\alpha }}S_{i}y_{i}^{-\alpha }}{1-e^{-\beta_{2}y_{i}^{-\alpha }}}=0 \end{array}$$ (11)

It is noted that both Eqs 10 and 11 do not yield explicit forms. Therefore, we apply numerical methods to find $\beta_{1_{MLE}}$ and $\beta_{2_{MLE}}$ and hence, *θ*_*MLE*_.

### Maximum product of spacing estimation

The MPSE method was first introduced by [19, 20] for estimating the parameters of continuous uni-variate distributions. They introduced the MPSE method as an alternative to the MLE approach by replacing the likelihood function with a product of spacing. They also showed that MPSE method has the same asymptotic properties as the MLE in the case where the density function’s support limits are known and when the density function’s support limits are unknown, MPSE method has better properties than the MLE.

[21] showed that the numerical behaviour of the MPSE is better than that of the likelihood and it can replace the likelihood function in Bayesian inference.

Many recent studies have been done on the MPSE method. See, for example, [22–24].

According to the model proposed by [19, 20] and the adaptive model by [25], the joint product spacing under adaptive type-II hybrid progressive censoring, ignoring the constant term, can be given by Eq 12:

$$\begin{array}{c} L_{\text{MPSE}}=\prod_{i=1}^{m_{1}+1}D_{i}\prod_{i=1}^{J_{1}}{\left(1-F_{1}\left(x_{i}\right)\right)}^{R_{i}}{\left(1-F_{1}\left(x_{m_{1}}\right)\right)}^{R_{m_{1}}}\prod_{i=1}^{m_{2}+1}C_{i}\prod_{i=1}^{J_{2}}{\left(1-F_{2}\left(y_{i}\right)\right)}^{S_{i}}{\left(1-F_{2}\left(y_{m_{2}}\right)\right)}^{S_{m_{2}}} \end{array}$$ (12)

where,

$$\begin{array}{c} D_{i}=\{\begin{array}{c} D_{1}=F_{1}\left(x_{1}\right);\;i=1\\ D_{i}=F_{1}\left(x_{i}\right)-F_{1}\left(x_{i-1}\right);\;i=2,\ldots ,m_{1}\\ D_{m_{1}+1}=1-F_{1}\left(x_{m_{1}}\right);\;i=m_{1}+1 \end{array} \end{array}$$

and

$$\begin{array}{c} C_{i}=\{\begin{array}{c} C_{1}=F_{2}\left(y_{1}\right);\;i=1\\ C_{i}=F_{2}\left(y_{i}\right)-F_{2}\left(y_{i-1}\right);\;i=2,\ldots ,m_{2}\\ C_{k+1}=1-F_{2}\left(y_{m_{2}}\right);\;i=m_{2}+1 \end{array} \end{array}$$

such that $\sum_{i=1}^{m_{1}+1}D_{i}=1$ and $\sum_{i=1}^{m_{2}+1}C_{i}=1$.

It is important to point out that if two observations are repeated, i.e., *x*_*i*_ = *x*_*i*−1_, the spacing *D*_*i*_ would be zero. In this case, one should substitute *f*(*x*_*i*_) for *D*_*i*_ in Eq 12 as suggested by [20].

Based on Eq 12, the likelihood function is given by Eq 13

$$\begin{array}{c} \begin{array}{cc} L_{\text{MPSE}}= & e^{-\beta_{1}x_{1}^{-\alpha }}\left(1-e^{-\beta_{1}x_{m_{1}}^{-\alpha }}\right)\prod_{i=2}^{m_{1}}\left(e^{-\beta_{1}x_{i}^{-\alpha }}-e^{-\beta_{1}x_{i-1}^{-\alpha }}\right)\prod_{i=1}^{J_{1}}{\left(1-e^{-\beta_{1}x_{i}^{-\alpha }}\right)}^{R_{i}}{\left(1-e^{-\beta_{1}x_{m_{1}}^{-\alpha }}\right)}^{R_{m_{1}}}\\ & e^{-\beta_{2}y_{1}^{-\alpha }}\left(1-e^{-\beta_{2}y_{m_{2}}^{-\alpha }}\right)\prod_{i=2}^{m_{2}}\left(e^{-\beta_{2}y_{i}^{-\alpha }}-e^{-\beta_{2}y_{i-1}^{-\alpha }}\right)\prod_{i=1}^{J_{2}}{\left(1-e^{-\beta_{2}y_{i}^{-\alpha }}\right)}^{S_{i}}{\left(1-e^{-\beta_{2}y_{m_{2}}^{-\alpha }}\right)}^{S_{m_{2}}} \end{array} \end{array}$$ (13)

The log of Eq 13 is given by Eq 14

$$\begin{array}{c} \begin{array}{cc} l_{MPSE}= & -\beta_{1}x_{1}^{-\alpha }+\text{ln}\left(1-e^{-\beta_{1}x_{m_{1}}^{-\alpha }}\right)+\sum_{i=2}^{m_{1}}\;\text{ln}\left(e^{-\beta_{1}x_{i}^{-\alpha }}-e^{-\beta_{1}x_{i-1}^{-\alpha }}\right)+\sum_{i=1}^{J_{1}}R_{i}\;\text{ln}\left(1-e^{-\beta_{1}x_{i}^{-\alpha }}\right)\\ & +R_{m_{1}}\;\text{ln}\left(1-e^{-\beta_{1}x_{m_{1}}^{-\alpha }}\right)-\beta_{2}y_{1}^{-\alpha }+\text{ln}\left(1-e^{-\beta_{2}y_{m_{2}}^{-\alpha }}\right)+\sum_{i=2}^{m_{2}}\;\text{ln}\left(e^{-\beta_{2}y_{i}^{-\alpha }}-e^{-\beta_{2}y_{i-1}^{-\alpha }}\right)\\ & +\sum_{i=1}^{J_{2}}S_{i}\;\text{ln}\left(1-e^{-\beta_{2}y_{i}^{-\alpha }}\right)+S_{m_{2}}\;\text{ln}\left(1-e^{-\beta_{2}y_{m_{2}}^{-\alpha }}\right) \end{array} \end{array}$$ (14)

To obtain the normal equations for the unknown parameters, we differentiate Eq 14 partially with respect to the scale parameters *β*_1_ and *β*_2_ and equate them to zero. The estimators $\beta_{1_{\text{MPSE}}}$ and $\beta_{2_{\text{MPSE}}}$ can be obtained by solving Eqs 15 and 16

$$\begin{array}{c} \begin{array}{cc} \frac{\partial \;l_{MPSE}}{\partial \beta_{1}}= & -x_{1}^{-\alpha }+\frac{x_{m_{1}}^{-\alpha }e^{-\beta_{1}x_{m_{1}}^{-\alpha }}}{1-e^{-\beta_{1}x_{m_{1}}^{-\alpha }}}+\frac{R_{m_{1}}x_{m_{1}}^{-\alpha }e^{-\beta_{1}x_{m_{1}}^{-\alpha }}}{1-e^{-\beta_{1}x_{m_{1}}^{-\alpha }}}+\sum_{i=1}^{J_{1}}\frac{R_{i}x_{i}^{-\alpha }e^{-\beta_{1}x_{i}^{-\alpha }}}{1-e^{-\beta_{1}x_{i}^{-\alpha }}}\\ & +\sum_{i=2}^{m_{1}}\frac{x_{i-1}^{-\alpha }e^{-\beta_{1}x_{i-1}^{-\alpha }}-x_{i}^{-\alpha }e^{-\beta_{1}}x_{i}^{-\alpha }}{-e^{-\beta_{1}x_{i-1}^{-\alpha }}+e^{-\beta_{1}x_{i}^{-\alpha }}}=0 \end{array} \end{array}$$ (15)

$$\begin{array}{c} \begin{array}{cc} \frac{\partial \;l_{MPSE}}{\partial \beta_{2}}= & -y_{1}^{-\alpha }+\frac{y_{m_{2}}^{-\alpha }e^{-\beta_{2}y_{m_{2}}^{-\alpha }}}{1-e^{-\beta_{2}y_{m_{2}}^{-\alpha }}}+\frac{S_{m_{2}}y_{m_{2}}^{-\alpha }e^{-\beta_{2}y_{m_{2}}^{-\alpha }}}{1-e^{-\beta_{2}y_{m_{2}}^{-\alpha }}}+\sum_{i=1}^{J_{2}}\frac{S_{i}y_{i}^{-\alpha }e^{-\beta_{2}y_{i}^{-\alpha }}}{1-e^{-\beta_{2}y_{i}^{-\alpha }}}\\ & +\sum_{i=2}^{m_{2}}\frac{y_{i-1}^{-\alpha }e^{-\beta_{2}y_{i-1}^{-\alpha }}-y_{i}^{-\alpha }e^{-\beta_{2}}y_{i}^{-\alpha }}{-e^{-\beta_{2}y_{i-1}^{-\alpha }}+e^{-\beta_{2}y_{i}^{-\alpha }}}=0 \end{array} \end{array}$$ (16)

The nonlinear equations Eqs 15 and 16 can’t be solved analytically, so $\beta_{1_{\text{MPSE}}}$ and $\beta_{2_{\text{MPSE}}}$ can be obtained using numerical methods, and hence the MPSE of *θ* can be obtained as follows $\theta_{\text{MPSE}}=\frac{\beta_{1_{\text{MPSE}}}}{\beta_{1_{\text{MPSE}}}+\beta_{2_{\text{MPSE}}}}$.

## Simulation study

### Simulation criteria

In this section we test, by simulation, the considered estimates of the SSR model under adaptive type-II hybrid progressive censoring. The following steps are used to find estimates of the stress and strength parameter *θ* using Mote Carlo Simulation from adaptive progressive type-II hybrid censored data at stopping time *T* using the method suggested by [2].

1. Generate two independent type-II censored samples $X_{1:m_{1}:n_{1}},X_{2:m_{1}:n_{1}},\ldots ,X_{m_{1}:m_{1}:n_{1}}$ and $Y_{1:m_{2}:n_{2}},Y_{2:m_{2}:n_{2}},\ldots ,Y_{m_{2}:m_{2}:n_{2}}$ from the IWD with shape parameter *α* and scale parameters *β*_1_ and *β*_2_ respectively with censoring schemes $\left(R_{1},R_{2},\ldots ,R_{m_{1}}\right)$ and $\left(S_{1},S_{2},\ldots ,S_{m_{2}}\right)$ as proposed by [26].
2. Determine the values of *J*_1_ and *J*_2_, such that $X_{J_{1}:m_{1}:n_{1}}<T_{1}<X_{J_{1}+1:m_{1}:n_{1}}$ and $Y_{J_{2}:m_{2}:n_{2}}<T_{2}<Y_{J_{2}+1:m_{2}:n_{2}}$, then remove $X_{J_{1}+2},\ldots ,X_{m_{1}:m_{1}:n_{1}}$ and $Y_{J_{2}+2},\ldots ,Y_{m_{2}:m_{2}:n_{2}}$.
3. Generate the first *m*_1_ − *j*_1_ − 1 order statistics from the truncated distribution $\frac{f(x)}{1-F(x_{J_{1}+1:m_{1}:n_{1}})}$ as $X_{J_{1}+2:m_{1}:n_{1}},\ldots ,X_{m_{1}:m_{1}:n_{1}}$ and the censoring scheme will change to $\left(R_{1},\ldots ,R_{J_{1}},0,\ldots ,0,R_{m_{1}}=n_{1}-m_{1}-\sum_{i=1}^{J_{1}}R_{i}\right)$. Similarly, generate the first *m*_2_−*j*_2_ − 1 order statistics from the truncated distribution $\frac{f(y)}{1-F(y_{J_{2}+1:m_{2}:n_{2}})}$ as $Y_{J_{2}+2:m_{2}:n_{2}},\ldots ,Y_{m_{2}:m_{2}:n_{2}}$ and the associated censoring scheme will change to $\left(S_{1},\ldots ,S_{J_{2}},0,\ldots ,0,S_{m_{2}}=n_{2}-m_{2}-\sum_{i=1}^{J_{2}}S_{i}\right)$
4. Calculate estimates of the scale parameters *β*_1_ and *β*_2_. The MLE and MPSE are both calculated using Newton Raphson method.
5. Calculate $\hat{\theta }$ using Eq 6).
6. After 3000 replications, calculate the Bias and the MSE to compare the estimated *θ* ($\hat{\theta }$) with the exact value of the previously determined SSR parameter *θ*_*exact*_ for each estimation method as follows:
  - Bias = $\left|{\bar{\theta }}_{i}-\theta_{exact}\right|$, where ${\bar{\theta }}_{i}$ is the average of the 3000 values of *θ*_*i*_ for both estimates.
  -
$\text{MSE}=\frac{\sum_{i=1}^{3000}{\left(\hat{\theta_{i}}-\theta_{exact}\right)}^{2}}{3000}$

In this article, the simulation has been performed by considering the shape parameter *α* = 1.5, without loss of generality, and three cases for *θ*_*exact*_ namely; 0.4, 0.6, and 0.9.

Three main stopping times are considered in this study that are chosen to be in three different time points during the experiment: $T_{1}=X_{\left\lfloor \frac{m}{4}\right\rfloor }$,$T_{2}=X_{\left\lfloor \frac{4m}{5}\right\rfloor }$, and after the failure of the last unit *T*_3_ = *X*_*m*_+ 2, in this case the adaptive censoring will be type-II progressive censoring. Three censoring schemes (C.s) are used in the simulation:

- C.s 1: $\{n-m{,0}^{*(m-1)}\}$, which is known as First-step censoring, i.e., n-m units are removed just after the first failure
- C.s 2: $\{0^{*(m-1)},n-m\}$, which is known as Right censoring, i.e., n-m units are removed after the last failure
- C.s 3: $\left\{\frac{n-m}{2}{,0}^{*(m-2)},\frac{n-m}{2}\right\}$ When removals take place at the beginning and at the end of the experiment, i.e., $\frac{n-m}{2}$ units are withdrawn just after the first failure and after the last failure

For brevity, we use the notation 0^*k^ to denote k successive zeros. Thus, the scheme {9, 0, 0, 0, 0, 0} is denoted by {9, 0^*5^}.

The sample sizes of the strength and stress components are chosen to be *n* = *n*_1_ = *n*_2_ = {20, 40, 60, 100} and the values of the effective sample sizes *m*_1_, *m*_2_ are chosen with a ratio of 0.2 and 0.5 of the sample sizes, i.e., when *n* = 20, m = 4 with a ratio of 0.2 and m = 10 with a ratio of 0.5, etc. Results of the simulation are summarized in Tables 1–9 as follows:

- Tables 1–3 provide the estimates at three stopping times and three censoring schemes when *θ* = 0.4.
- Tables 4–6 provide the estimates at three stopping times and three censoring schemes when *θ* = 0.6.
- Tables 7–9 provide the estimates at three stopping times and three censoring schemes when *θ* = 0.9.

**Table 1:** Bias and MSE of *θ* with ${\text{T}}_{1}={\text{X}}_{\frac{m}{4}}$ for different censoring schemes when *θ* = 0.4..

n,m	C.s	Bias		MSE		n,m	C.s	Bias		MSE	
		MLE	MPSE	MLE	MPSE			MLE	MPSE	MLE	MPSE
20,4	1	0.00732	0.00712	0.00774	0.00758	60,18	1	0.00083	0.00078	0.00232	0.00230
	2	0.00262	0.00261	0.00557	0.00545		2	0.00091	0.00090	0.00139	0.00138
	3	0.00370	0.00352	0.00657	0.00634		3	0.00011	0.00014	0.00170	0.00167
20,10	1	0.00252	0.00240	0.00414	0.00406	60,30	1	0.00055	0.00056	0.00148	0.00147
	2	0.00015	0.00011	0.00351	0.00344		2	0.00039	0.00045	0.00112	0.00111
	3	0.00232	0.00234	0.00389	0.00381		3	0.00072	0.00066	0.00132	0.00131
40,12	1	0.00051	0.00050	0.00310	0.00305	100,20	1	0.00112	0.00112	0.00190	0.00189
	2	0.00026	0.00025	0.00217	0.00213		2	0.00083	0.00084	0.00104	0.00103
	3	0.00068	0.00057	0.00248	0.00242		3	0.00175	0.00172	0.00134	0.00132
40,20	1	0.00145	0.00135	0.00216	0.00214	100,50	1	0.00152	0.00149	0.00091	0.00091
	2	0.00177	0.00177	0.00175	0.00173		2	0.00011	0.00008	0.00063	0.00063
	3	0.00012	0.00010	0.00192	0.00190		3	0.00029	0.00029	0.00074	0.00073

**Table 2:** Bias and MSE of *θ* with ${\text{T}}_{1}={\text{X}}_{\frac{4m}{5}}$ for different censoring schemes when *θ* = 0.4.

n,m	C.s	Bias		MSE		n,m	C.s	Bias		MSE	
		MLE	MPSE	MLE	MPSE			MLE	MPSE	MLE	MPSE
20,4	1	0.00315	0.00305	0.00699	0.00685	60,18	1	0.00158	0.00161	0.00230	0.00229
	2	0.00317	0.00307	0.00548	0.00537		2	0.00156	0.00155	0.00138	0.00137
	3	0.00484	0.00467	0.00635	0.00616		3	0.00157	0.00154	0.00163	0.00160
20,10	1	0.00312	0.00308	0.00440	0.00432	60,30	1	0.00158	0.00161	0.00230	0.00229
	2	0.00274	0.00275	0.00338	0.00331		2	0.00156	0.00155	0.00138	0.00137
	3	0.00367	0.00364	0.00380	0.00372		3	0.00157	0.00154	0.00163	0.00160
40,12	1	0.00061	0.00064	0.00315	0.00310	100,20	1	0.00112	0.00119	0.00189	0.00188
	2	0.00010	0.00011	0.00212	0.00209		2	0.00014	0.00016	0.00098	0.00097
	3	0.00159	0.00161	0.00241	0.00237		3	0.00131	0.00128	0.00126	0.00124
40,20	1	0.00128	0.00132	0.00222	0.00220	100,50	1	0.00008	0.00010	0.00092	0.00092
	2	0.00027	0.00027	0.00168	0.00166		2	0.00122	0.00121	0.00064	0.00064
	3	0.00079	0.00086	0.00191	0.00189		3	0.00115	0.00116	0.00077	0.00077

**Table 3:** Bias and MSE of *θ* with T_3_ = X_m_ + 2 for different censoring schemes when *θ* = 0.4.

n,m	C.s	Bias		MSE		n,m	C.s	Bias		MSE	
		MLE	MPSE	MLE	MPSE			MLE	MPSE	MLE	MPSE
20,4	1	0.00418	0.00414	0.00690	0.00676	60,18	1	0.00172	0.00175	0.00220	0.00220
	2	0.00261	0.00255	0.00541	0.00529		2	0.00037	0.00037	0.00138	0.00136
	3	0.00228	0.00229	0.00645	0.00625		3	0.00087	0.00089	0.00176	0.00173
20,10	1	0.00230	0.00235	0.00419	0.00412	60,30	1	0.00137	0.00138	0.00146	0.00146
	2	0.00095	0.00099	0.00339	0.00332		2	0.00027	0.00028	0.00109	0.00108
	3	0.00363	0.00356	0.00380	0.00373		3	0.00156	0.00158	0.00136	0.00135
40,12	1	0.00146	0.00139	0.00318	0.00313	100,20	1	0.00058	0.00068	0.00197	0.00197
	2	0.00192	0.00190	0.00224	0.00221		2	0.00067	0.00065	0.00097	0.00096
	3	0.00065	0.00058	0.00254	0.00249		3	0.00010	0.00012	0.00127	0.00126
40,20	1	0.00098	0.00104	0.00218	0.00216	100,50	1	0.00106	0.00107	0.00092	0.00092
	2	0.00092	0.00089	0.00178	0.00175		2	0.00011	0.00008	0.00064	0.00063
	3	0.00122	0.00118	0.00196	0.00193		3	0.00002	0.00001	0.00073	0.00072

**Table 4:** Bias and MSE of *θ* with ${\text{T}}_{1}={\text{X}}_{\frac{m}{4}}$ for different censoring schemes when *θ* = 0.6.

n,m	C.s	Bias		MSE		n,m	C.s	Bias		MSE	
		MLE	MPSE	MLE	MPSE			MLE	MPSE	MLE	MPSE
20,4	1	0.00301	0.00295	0.00740	0.00726	60,18	1	0.00229	0.00228	0.00217	0.00215
	2	0.00134	0.00132	0.00552	0.00540		2	0.00054	0.00049	0.00136	0.00135
	3	0.00513	0.00496	0.00624	0.00604		3	0.00018	0.00017	0.00171	0.00168
20,10	1	0.00179	0.00184	0.00436	0.00427	60,30	1	0.00110	0.00110	0.00147	0.00147
	2	0.00218	0.00213	0.00355	0.00347		2	0.00015	0.00015	0.00112	0.00111
	3	0.00184	0.00190	0.00389	0.00382		3	0.00066	0.00068	0.00125	0.00124
40,12	1	0.00064	0.00055	0.00335	0.00330	100,20	1	0.00020	0.00024	0.00193	0.00192
	2	0.00195	0.00187	0.00220	0.00216		2	0.00030	0.00029	0.00103	0.00102
	3	0.00125	0.00124	0.00248	0.00243		3	0.00119	0.00118	0.00126	0.00125
40,20	1	0.00179	0.00170	0.00212	0.00210	100,50	1	0.00021	0.00021	0.00089	0.00089
	2	0.00102	0.00101	0.00180	0.00177		2	0.00069	0.00070	0.00063	0.00062
	3	0.00006	0.00008	0.00130	0.00128		3	0.00006	0.00008	0.00077	0.00077

**Table 5:** Bias and MSE of *θ* with ${\text{T}}_{2}={\text{X}}_{\frac{4m}{5}}$ for different censoring schemes when *θ* = 0.6.

n,m	C.s	Bias		MSE		n,m	C.s	Bias		MSE	
		MLE	MPSE	MLE	MPSE			MLE	MPSE	MLE	MPSE
20,4	1	0.00261	0.00262	0.00719	0.00704	60,18	1	0.00100	0.00097	0.00229	0.00227
	2	0.00034	0.00027	0.00556	0.00543		2	0.00077	0.00076	0.00140	0.00138
	3	0.00485	0.00463	0.00632	0.00613		3	0.00025	0.00023	0.00175	0.00172
20,10	1	0.00074	0.00070	0.00421	0.00413	60,30	1	0.00019	0.00024	0.00149	0.00148
	2	0.00200	0.00197	0.00341	0.00334		2	0.00112	0.00112	0.00111	0.00110
	3	0.00232	0.00222	0.00383	0.00375		3	0.00093	0.00094	0.00128	0.00127
40,12	1	0.00049	0.00057	0.00338	0.00334	100,20	1	0.00074	0.00075	0.00187	0.00187
	2	0.00232	0.00229	0.00217	0.00214		2	0.00127	0.00125	0.00106	0.00105
	3	0.00170	0.00174	0.00254	0.00249		3	0.00033	0.00032	0.00130	0.00128
40,20	1	0.00122	0.00119	0.00221	0.00219	100,50	1	0.00118	0.00122	0.00096	0.00096
	2	0.00038	0.00036	0.00172	0.00169		2	0.00069	0.00067	0.00067	0.00067
	3	0.00189	0.00185	0.00185	0.00182		3	0.00043	0.00044	0.00079	0.00079

**Table 6:** Bias and MSE of *θ* with T_3_ = X_m_ + 2 for different censoring schemes when *θ* = 0.6.

n,m	C.s	Bias		MSE		n,m	C.s	Bias		MSE	
		MLE	MPSE	MLE	MPSE			MLE	MPSE	MLE	MPSE
20,4	1	0.00173	0.00170	0.00721	0.00707	60,18	1	0.00154	0.00139	0.00217	0.00216
	2	0.00216	0.00206	0.00576	0.00562		2	0.00093	0.00094	0.00138	0.00136
	3	0.00509	0.00493	0.00640	0.00619		3	0.00247	0.00240	0.00170	0.00167
20,10	1	0.00299	0.00286	0.00426	0.00418	60,30	1	0.00016	0.00018	0.00157	0.00156
	2	0.00037	0.00032	0.00350	0.00343		2	0.00003	0.00001	0.00108	0.00108
	3	0.00247	0.00240	0.00388	0.00379		3	0.00164	0.00164	0.00129	0.00127
40,12	1	0.00339	0.00336	0.00339	0.00333	100,20	1	0.00038	0.00039	0.00191	0.00494
	2	0.00013	0.00010	0.00220	0.00217		2	0.00107	0.00108	0.00103	0.00102
	3	0.00249	0.00246	0.00256	0.00251		3	0.00082	0.00081	0.00132	0.00130
40,20	1	0.00145	0.00149	0.00221	0.00219	100,50	1	0.00110	0.00113	0.00087	0.00088
	2	0.00026	0.00028	0.00170	0.00167		2	0.00082	0.00081	0.00068	0.00067
	3	0.00078	0.00071	0.00185	0.00182		3	0.00000	0.00002	0.00078	0.00077

**Table 7:** Bias and MSE of *θ* with ${\text{T}}_{1}={\text{X}}_{\frac{m}{4}}$ for different censoring schemes when *θ* = 0.9.

n,m	C.s	Bias		MSE		n,m	C.s	Bias		MSE	
		MLE	MPSE	MLE	MPSE			MLE	MPSE	MLE	MPSE
20,4	1	0.00448	0.00438	0.00122	0.00119	60,18	1	0.00153	0.00149	0.00033	0.00033
	2	0.00400	0.00391	0.00092	0.00089		2	0.00112	0.00111	0.00022	0.00021
	3	0.00445	0.00428	0.00101	0.00097		3	0.00115	0.00114	0.00025	0.00025
20,10	1	0.00275	0.00270	0.00068	0.00066	60,30	1	0.00083	0.00084	0.00022	0.00022
	2	0.00169	0.00163	0.00053	0.00050		2	0.00076	0.00073	0.00016	0.00016
	3	0.00270	0.00263	0.00062	0.00061		3	0.00101	0.00100	0.00019	0.00018
40,12	1	0.00174	0.00172	0.00047	0.00047	100,20	1	0.00046	0.00046	0.00027	0.00028
	2	0.00147	0.00146	0.00032	0.00032		2	0.00060	0.00059	0.00015	0.00015
	3	0.00222	0.00214	0.00039	0.00036		3	0.00079	0.00078	0.00018	0.00018
40,20	1	0.00141	0.00136	0.00033	0.00032	100,50	1	0.00039	0.00039	0.00014	0.00014
	2	0.00120	0.00119	0.00025	0.00025		2	0.00078	0.00077	0.00010	0.00010
	3	0.00151	0.00149	0.00029	0.00028		3	0.00079	0.00079	0.00011	0.00011

**Table 8:** Bias and MSE of *θ* with ${\text{T}}_{2}={\text{X}}_{\frac{4m}{5}}$ for different censoring schemes when *θ* = 0.9.

n,m	C.s	Bias		MSE		n,m	C.s	Bias		MSE	
		MLE	MPSE	MLE	MPSE			MLE	MPSE	MLE	MPSE
20,4	1	0.00362	0.00354	0.00115	0.00112	60,18	1	0.00108	0.00104	0.00034	0.00033
	2	0.00369	0.00360	0.00090	0.00088		2	0.00074	0.00074	0.00020	0.00020
	3	0.00434	0.00422	0.00108	0.00104		3	0.00131	0.00128	0.00025	0.00025
20,10	1	0.00361	0.00357	0.00069	0.00068	60,30	1	0.00096	0.00097	0.00021	0.00021
	2	0.00234	0.00230	0.00055	0.00054		2	0.00069	0.00069	0.00016	0.00016
	3	0.00305	0.00305	0.00062	0.00045		3	0.00096	0.00096	0.00019	0.00018
40,12	1	0.00184	0.00185	0.00049	0.00048	100,20	1	0.00144	0.00144	0.00028	0.00028
	2	0.00150	0.00148	0.00032	0.00032		2	0.00061	0.00060	0.00015	0.00014
	3	0.00176	0.00172	0.00041	0.00041		3	0.00074	0.00072	0.00018	0.00017
40,20	1	0.00158	0.00157	0.00033	0.00033	100,50	1	0.00093	0.00095	0.00014	0.00013
	2	0.00104	0.00102	0.00024	0.00069		2	0.00032	0.00032	0.00010	0.00009
	3	0.00191	0.00188	0.00028	0.00028		3	0.00063	0.00063	0.00011	0.00011

**Table 9:** Bias and MSE of *θ* with T_3_ = X_m_+2 for different censoring schemes when *θ* = 0.9.

n,m	C.s	Bias		MSE		n,m	C.s	Bias		MSE	
		MLE	MPSE	MLE	MPSE			MLE	MPSE	MLE	MPSE
20,4	1	0.00555	0.00543	0.00118	0.00115	60,18	1	0.00127	0.00126	0.00033	0.00033
	2	0.00276	0.00270	0.00087	0.00085		2	0.00073	0.00071	0.00021	0.00021
	3	0.00321	0.00306	0.00098	0.00094		3	0.00133	0.00132	0.00025	0.00024
20,10	1	0.00275	0.00271	0.00066	0.00064	60,30	1	0.00088	0.00087	0.00022	0.00022
	2	0.00282	0.00277	0.00057	0.00056		2	0.00057	0.00056	0.00016	0.00016
	3	0.00250	0.00243	0.00060	0.00058		3	0.90309	0.00063	0.00018	0.00018
40,12	1	0.00229	0.00226	0.00049	0.00049	100,20	1	0.00080	0.00080	0.00028	0.00028
	2	0.00156	0.00154	0.00032	0.00032		2	0.00067	0.00067	0.00014	0.00014
	3	0.00169	0.00166	0.00038	0.00037		3	0.00067	0.00065	0.00018	0.00017
40,20	1	0.00142	0.00138	0.00032	0.00031	100,50	1	0.00092	0.00115	0.00014	0.00014
	2	0.00060	0.00058	0.00025	0.00025		2	0.00014	0.00013	0.00009	0.00009
	3	0.00130	0.00132	0.00028	0.00027		3	0.00077	0.00076	0.00011	0.00011

### Simulation results

Results are summarized as follows:

- The MPSE performs slightly better than the MLE for small sample sizes.
- The MPSE and MLE are roughly the same for large sample sizes.
- In general, Bias and MSE of the calculated estimates decrease as effective sample sizes increase.
- It is noted that the estimates perform better under the second censoring scheme where removals take place after the m-th failure.
- It is worth mentioning that estimates perform best when *θ* = 0.9 and results are quite similar when *θ* = 0.4 and 0.6 and this observation is also noted by [28], where the behavior of the reliability model is graphed with different sample sizes in relation to MSE, and it was concluded that when the stress and strength components have the same effective sample size the curve of the reliability model is symmetric with respect to 0.5, meaning that the estimates of *θ* have a higher MSE when the reliability tends to 0.5.

### Real-life examples

This section considers two real-life examples to illustrate both proposed methods and further apply our knowledge based on our simulation study.

**Example 1:** We consider two data sets by [28] representing the breakdown times (in minutes) of an insulating fluid between two electrodes recorded at different voltages; 34 kilo-volts (data I) and 36 Kilo-volts (data II), as presented in Table 10.

**Table 10:** Breakdown times (in minutes) for data I and data II.

**Data I**	0.19	0.78	0.96	1.31	2.78	3.16	4.15	4.67	4.85	6.5
	7.35	8.01	8.27	12.06	31.75	32.52	33.91	36.71	72.89	
**Data II**	0.35	0.59	0.96	0.99	1.69	1.97	2.07	2.58	2.71	2.9
	3.67	3.99	5.35	13.77	25.50					

Many Authors discussed this data, see for example: [29–32].

The estimated parameter values (*α*, *β*) of the fitted IWD PDF for data I were (0.7015,1.8886), and (1.0823,1.3309) for data II. To check the goodness of fit of the IWD for data sets I and II, three statistical tools are used: Kolmogorov-Smirnov test (K-S), Anderson-Darling test (A-D), and Chi-Squared test. The results are summarized in Table 11 with a significance level of 0.05.

**Table 11:** Test statistic and p-value associated with each test for example 1.

Data	K-S (p-value)	A-D (p-value)	Chi-Squared (p-value)
I	0.1873 (0.4625)	0.7723 (0.4986)	0.8865 (0.6420)
II	0.2037 (0.4991)	0.4929 (0.7509)	1.4421 (0.2298)

The p-value for each test is more than 0.05. Hence, the IWD is a good fit for both data sets I and II.

**Fig 6:**
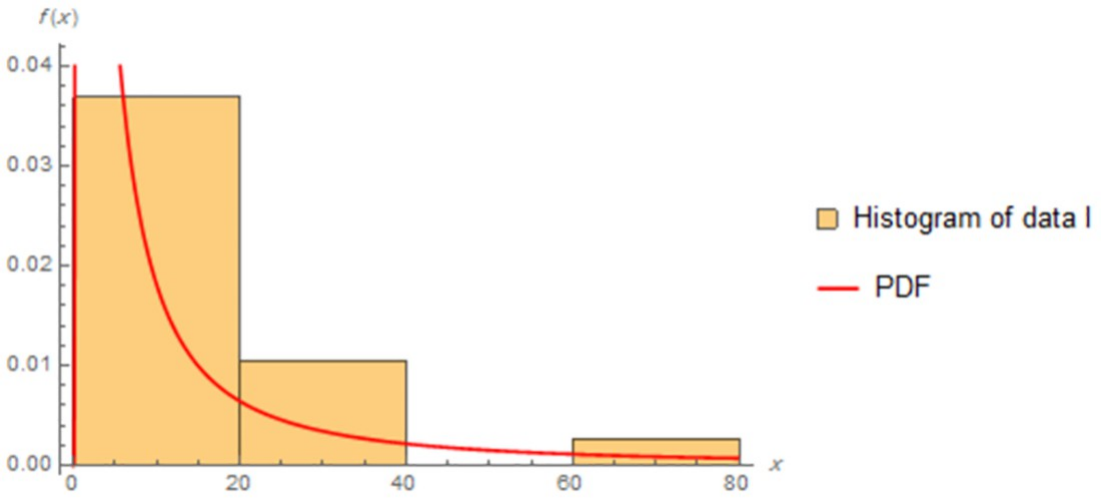
Estimated PDF of data I.

The fitted PDFs and Q-Q plots are plotted for both data sets as shown in Figs 6 and 9.

**Fig 7:**
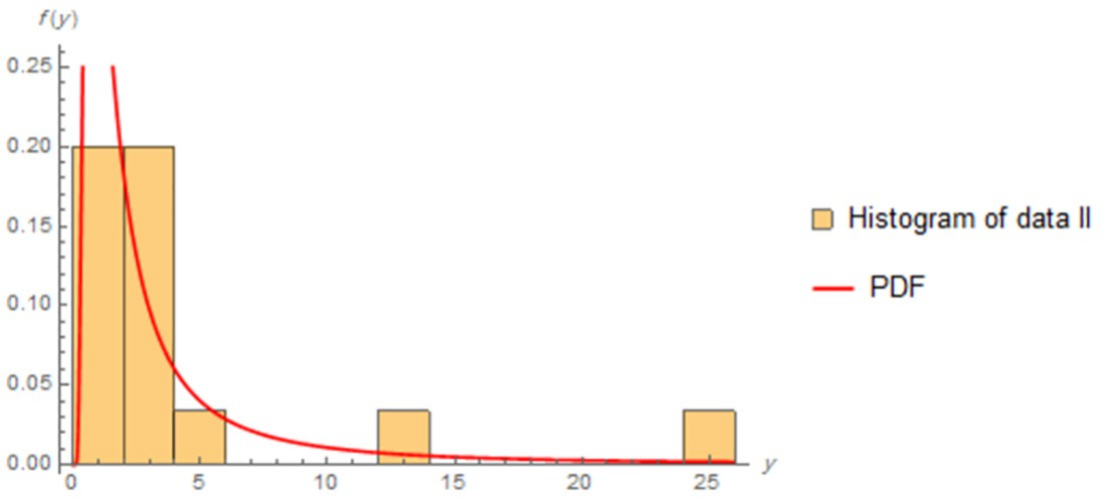
Estimated PDF of data II.

**Fig 8:**
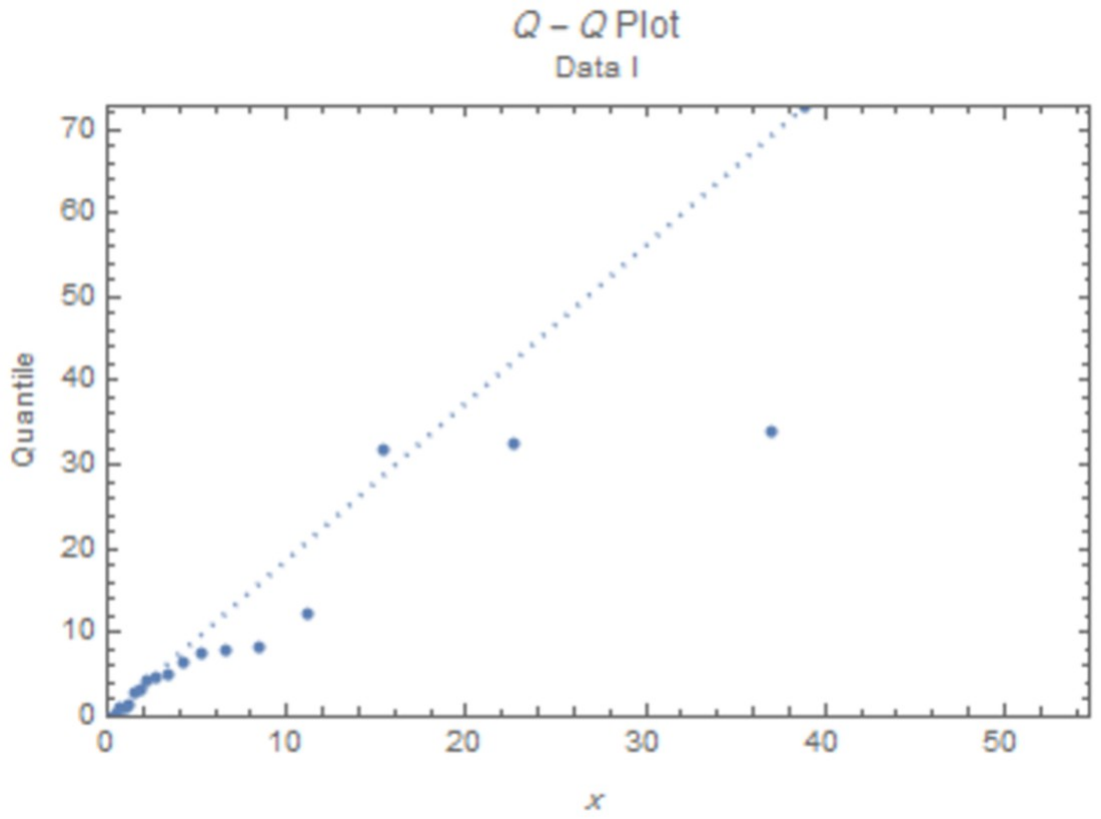
Q-Q plot for data I.

**Fig 9:**
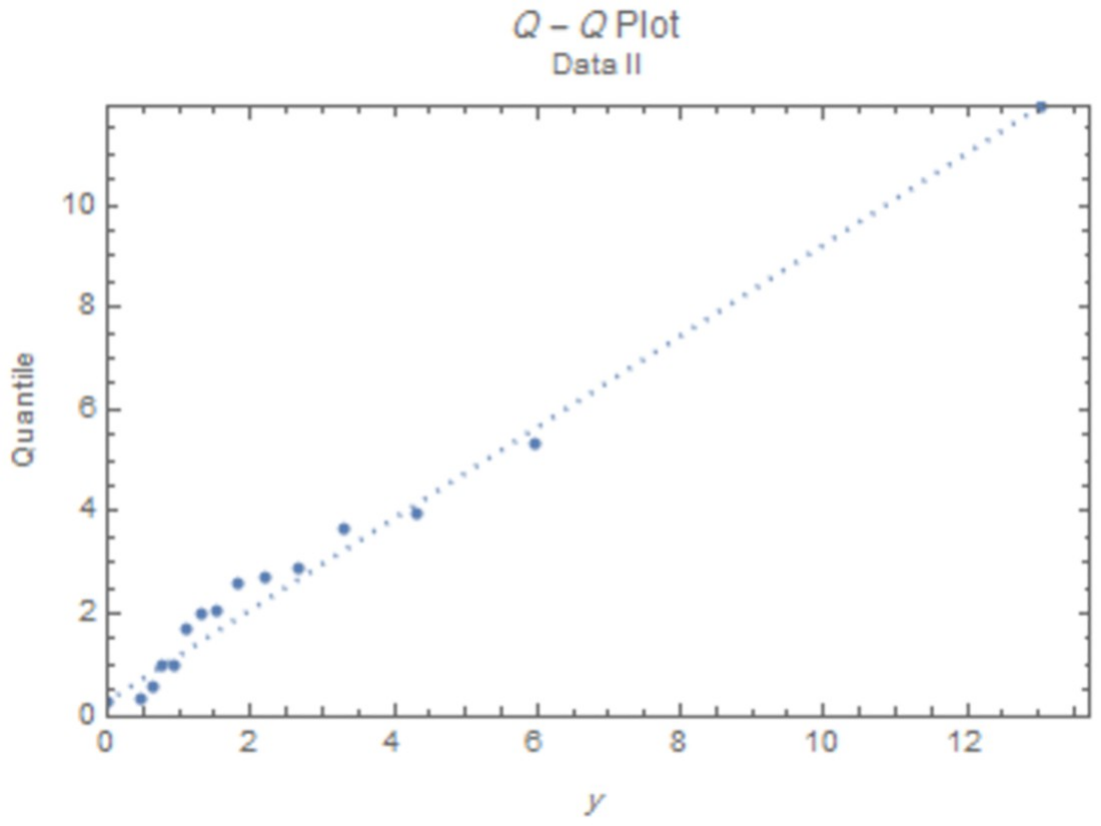
Q-Q plot for data II.

Figs 6–9 show that the estimated PDF of the IWD is a good fit for both data sets I and II.

The three adaptive type-II censoring schemes for the simulation study are used to generate adaptive type-II hybrid progressive censored samples, the associated stopping time for each scheme and the generated censored samples are given in Table 12.

**Table 12:** Adaptive type-II censored samples form data I and data II.

**C.s**	**T_1_**	**Censored sample from data I**										
1	2	0.19	0.78	1.31	2.78	3.16	4.67	7.35	8.01	12.06	31.75	32.52
2	8	0.19	0.78	0.96	1.31	2.78	3.16	4.15	4.67	4.85	6.5	7.35
3	7.5	0.19	0.78	1.31	3.16	4.15	4.67	4.85	7.35	8.01	12.06	31.75
**C.s**	**T_2_**	**Censored sample from data I**										
1	1	0.35	0.59	0.99	1.97	2.07	2.58	2.9	3.67	3.99		
2	3	0.35	0.59	0.96	0.99	1.69	1.97	2.07	2.58	2.71		
3	2	0.35	0.59	0.96	1.69	1.97	2.58	2.71	3.67	5.35		

The estimates of the SSR model are calculated for the complete case and the three censoring scheme with effective sample sizes m_1_ = 11, m_2_ = 9. Results are summarized in Table 13

**Table 13:** Estimates of *θ* for example 1.

C.s	Complete	1	2	3
MLE	0.5690	0.5626	0.5695	0.5714
MPSE	0.5726	0.5623	0.5731	0.5739

From Table 13, we can see that *θ* is more than 0.5 which means that data I has a higher probability of having longer survival times than data II. Moreover, estimates of *θ* based on the adaptive type-II hybrid progressive samples are close to those of complete data.

Furthermore, 95% Bootstrap confidence intervals are computed for the calculated estimators of *θ* as shown in Table 14.

**Table 14:** Bootstrap confidence intervals for the considered estimates of *θ* for example 1.

C.s	1	2	3
MLE	(0.3019, 0.807)	(0.383, 0.7388)	(0.3279, 0.7865)
MPSE	(0.2989, 0.8095)	(0.3837, 0.7419)	(0.3252, 0.7919)

From Table 14 we can see that all estimates of *θ* in Table 13 lie inside the bootstrap confidence intervals. Next, we calculate the standard error and average values resulted from bootstrapping for each estimate and results are in Table 15.

**Table 15:** Standard error and average value for each estimate after bootstrapping for example 1.

Standard error				Average value			
C.s	1	2	3	C.s	1	2	3
MLE	0.1302	0.0908	0.12	MLE	0.5668	0.5713	0.5688
MPSE	0.1312	0.0917	0.1204	MPSE	0.5677	0.574	0.5711

From Table 15, we note that the standard error is the least for most of the estimates of *θ* under the second censoring scheme. Moreover, Bayes estimates under LINEX loss function when λ = 1 have the lowest error under the second and third schemes. Average values of the estimates of *θ* are close to those in Table 13.

Example 2: We consider the data used by [33] of two groups of patients with head and neck cancer (HNC). Patients in one group were treated with radiotherapy (RT) and their survival times were recorded in days (Data 1) as follows; 7, 34, 42, 63, 64, **74**, 83, 84, 91, 108, 112, 119, 133, 133, 139, 140, 140, 146, 149, 154, 157, 160, 160, 165, 173, 176, **185**, 218, 225, 241, 248, 273, 277, **279**, 279, **319**, 405, 417, 420, 440, 523, **523**, 583, 594, 1101, **1116**, 1146, **1226**, **1349**, **1412**, 1417. Patients in the other group were treated with a combination of chemotherapy and radiotherapy (CT+RT) and their survival times were recorded in days (Data 2) as follows; 37, 84, 92, 94, 110, 112, 119, 127, 130, 133, 140, 146, 155, 159, **169**, 173, 179, 194, 195, 209, 249, 281, 319, 339, 432, 469, 519, **528**, **547**, **613**, 633, 725, **759**, 817, **1092**, **1245**, **1331**, 1557, **1642**, **1771**, 1776, **1897**, **2023**, **2146**, **2297** as reported by Efron (1988). Failure times in bold are censored observations that mainly represent patients that left the treatment center and never reported back. [33] analyzed the survival times of both data sets and concluded that the cubic linear model gives the best fit to the data compared with other models. He also compared the two therapies based on estimated survival functions under each model and found that CT + RT provides better HNC patient survival time than RT.

[34] used the truncated log-normal distribution to generate the unknown censored data in months by dividing the survival times by 30.438 to avoid overflow in large values while computing. The retrieved survival times in months for data 1 are 6.53, 10.42, 14.48, 16.1, 22.7, 41.55, 45.28, 49.4 and 53.62, for data 2 the retrieved survival times in months are 12.2, 23.56, 23.74, 25.87, 31.98, 41.35, 47.38, 55.46, 58.36, 63.74, 68.46, 78.26, 74.47, and 81.43. The survival times (in months) are illustrated in Table 16.

**Table 16:** Survival times (in months) for data 1 and data 2.

Data 1	0.23	1.12	1.38	2.07	2.10	2.73	2.76	2.99	3.55
	3.68	4.24	4.37	4.37	4.57	4.60	4.60	4.80	4.90
	5.06	5.16	5.26	5.26	5.42	5.68	5.78	6.53	7.16
	7.39	7.02	8.15	8.97	9.10	9.76	10.42	13.31	13.70
	13.80	14.46	14.48	16.10	17.18	19.15	19.52	22.70	36.17
	37.65	41.55	45.28	46.55	49.40	53.62			
Data 2	1.22	2.76	3.02	3.09	3.61	3.68	3.91	4.17	4.27
	4.37	4.60	4.80	5.09	5.22	5.68	5.88	6.37	6.41
	6.87	8.18	9.23	10.48	11.14	12.20	14.91	15.41	17.05
	20.80	23.56	23.74	23.82	25.87	26.84	31.98	41.35	47.38
	51.15	55.46	58.38	58.36	63.47	68.46	74.47	78.26	81.43

[33] noted a uni-modal behavior of the empirical hazard rate as obtained from the two data sets, based on that the IWD is a good candidate model for the two data sets. To test this assumption, we test the goodness of fit of the IWD for data sets I and II using K-S, A-D, and Chi-Squared tests. The results are summarized in Table 17 with a significance level of 0.05.

**Table 17:** Test statistic and p-value associated with each test for example 2.

Data	K-S (p-value)	A-D (p-value)	Chi-Squared (p-value)
1	0.1606 (0.1290)	1.383 (0.2065)	5.6975 (0.3368)
2	0.1175 (0.5248)	0.7716 (0.5003)	3.7435 (0.4418)

The p-value for each test is more than 0.05. Hence, the IWD is a good fit for both data sets 1 and 2.

From Table 17, we can clearly see that the p-value for each test is more than 0.05. Hence, the IWD is a good fit for both data sets 1 and 2.

**Fig 10:**
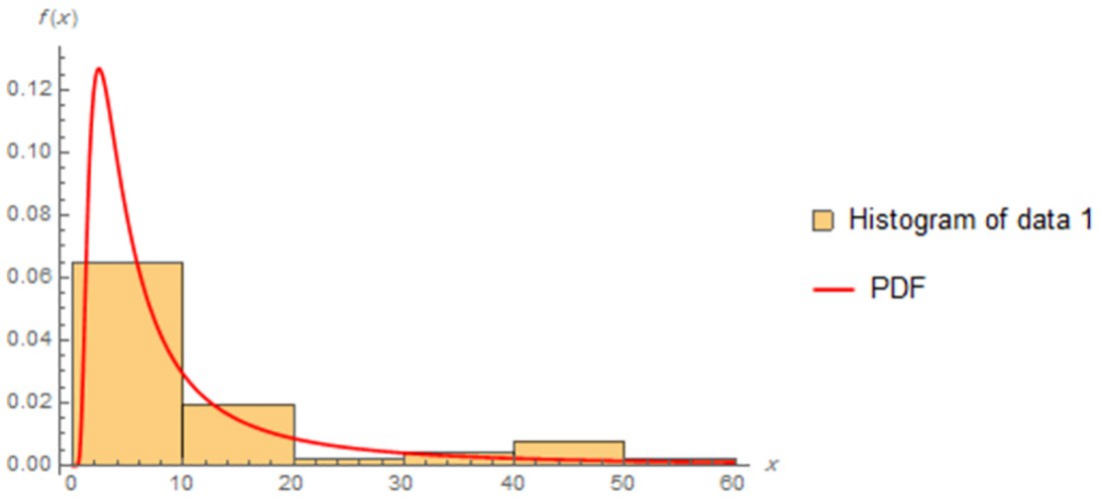
Estimated PDF of data 1.

**Fig 11:**
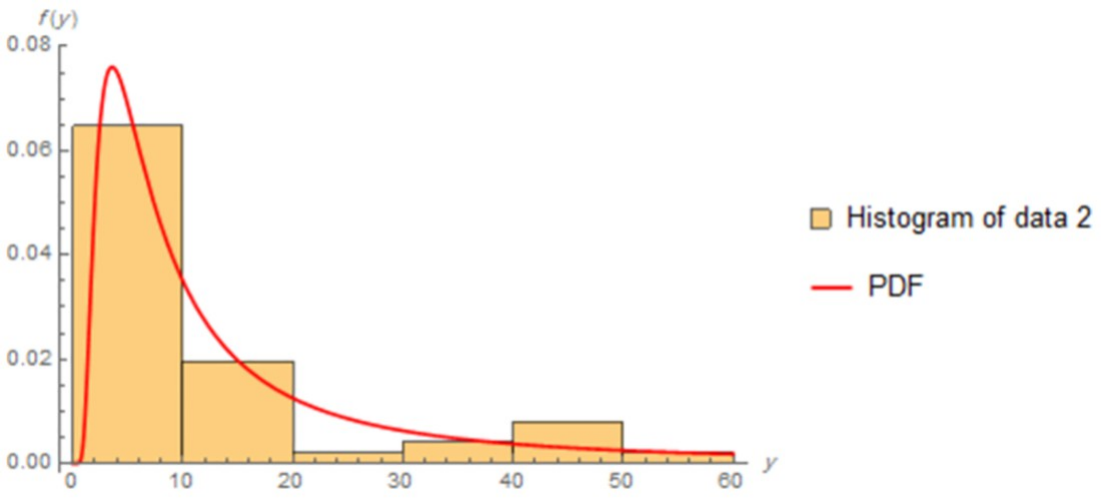
Estimated PDF of data 2.

**Fig 12:**
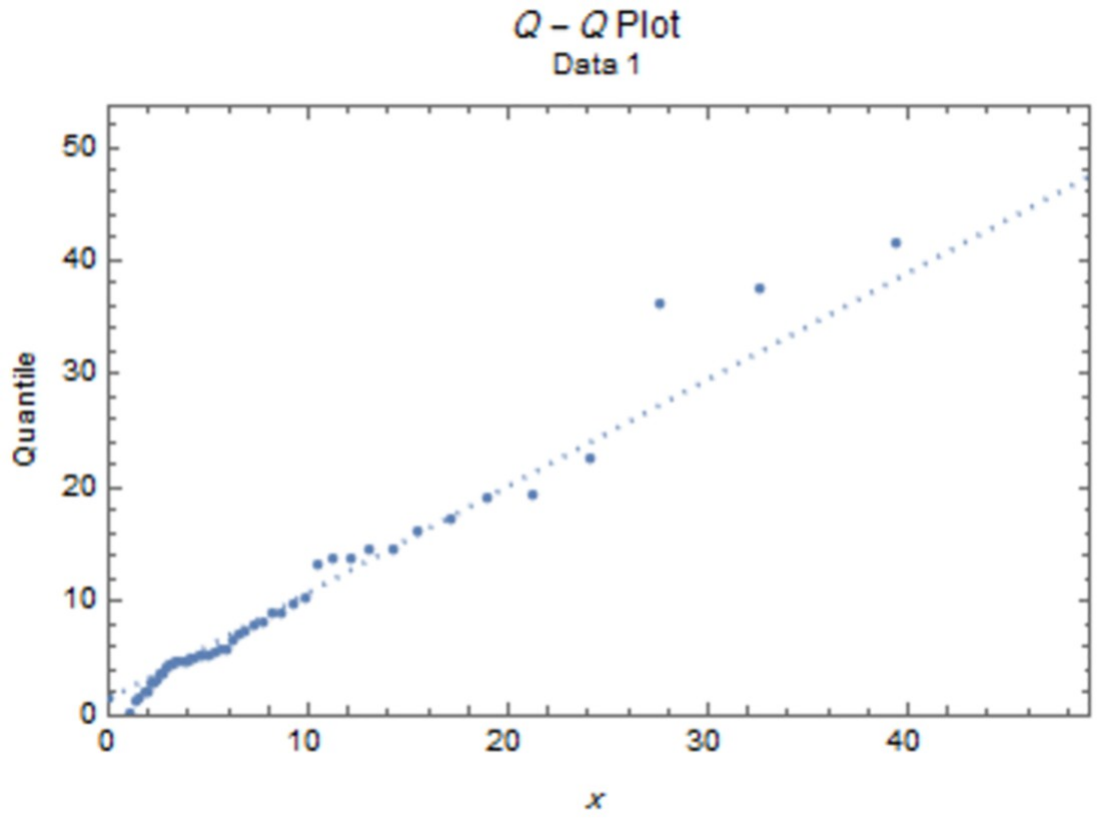
Q-Q plot for data 1.

**Fig 13:**
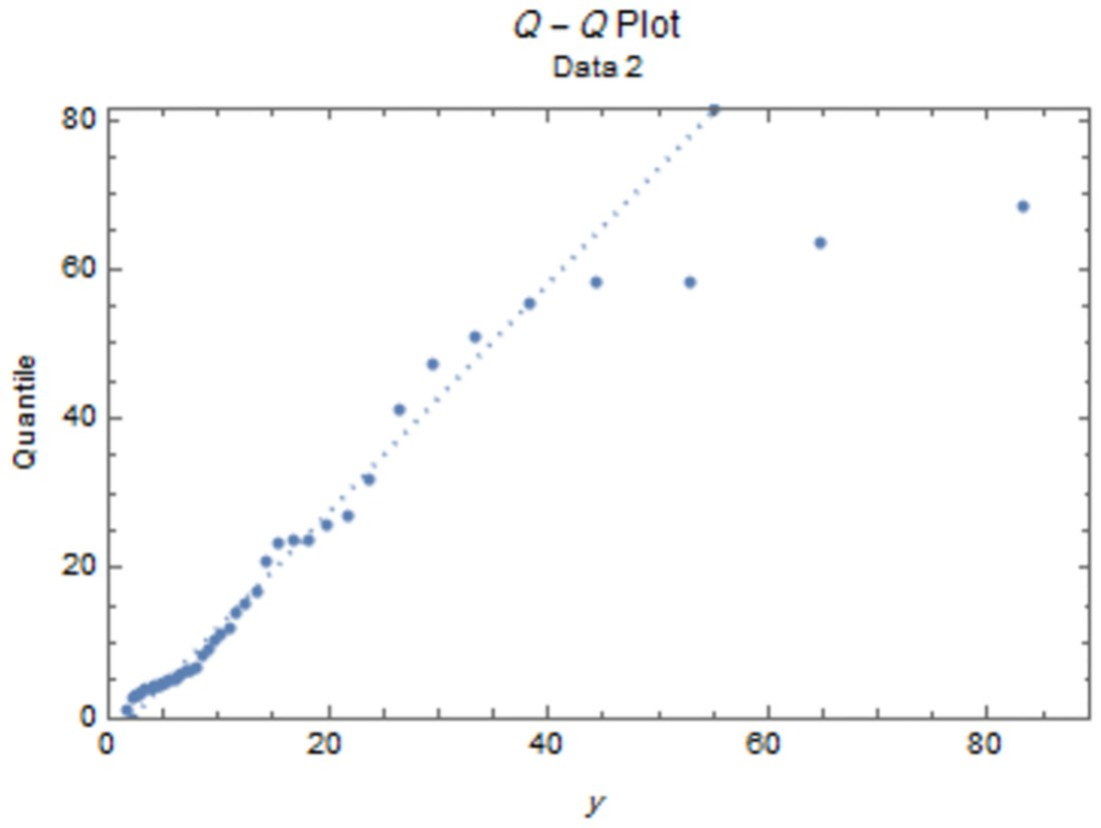
Q-Q plot for data 2.

The estimated parameter values of the fitted IWD PDF (*α*, *β*) for data 1 are (1.0657, 4.8044), and (1.0021,7.117) for data 2. The fitted PDFs and Q-Q plots are graphed for both data sets as shown in Figs 10–13.

Figs 10–13 show that the estimated PDF of the IWD is a good fit for both data sets 1 and 2.

The estimates of *θ* are calculated with effective sample sizes m_1_ = 26, m_2_ = 22. The three adaptive type-II progressive hybrid censoring schemes for the simulation study are used to generate adaptive type-II hybrid progressive censored samples, the associated stopping time for each scheme and the generated censored samples are given in Table 18.

**Table 18:** Adaptive type-II censored samples form data 1 and data 2.

Scheme	*T*_1_	Censored sample from data 1					
1	2.1	0.23	1.12	1.38	2.07	2.73	2.99
		3.55	3.68	4.24	4.37	4.60	4.60
		5.06	5.16	5.42	5.68	6.53	7.16
		7.92	9.76	10.42	13.31	13.80	14.46
		17.18	19.52				
2	7	0.23	1.12	1.38	2.07	2.10	2.73
		2.76	2.99	3.55	3.68	4.24	4.37
		4.37	4.57	4.60	4.60	4.80	4.90
		5.06	5.16	5.26	5.26	5.42	5.68
		5.78	6.53				
3	5	0.23	1.12	2.10	2.76	2.99	3.55
		3.68	4.24	4.37	4.60	5.06	5.26
		5.26	5.78	7.16	7.39	8.15	9.76
		10.42	13.31	13.80	14.46	17.18	19.15
		19.52	22.70				
Scheme	*T*_2_	Censored sample from data 2					
1	3.5	1.22	2.76	3.02	3.61	4.17	4.37
		4.60	5.22	5.68	6.37	8.18	9.23
		10.48	12.20	14.91	17.05	20.80	23.82
		26.84	31.98	41.35	47.38		
2	11	1.22	2.76	3.02	3.09	3.61	3.68
		3.91	4.17	4.27	4.37	4.60	4.80
		5.09	5.22	5.68	5.88	6.37	6.41
		6.87	8.18	9.23	10.48		
3	9	1.22	2.76	3.09	3.68	4.17	4.27
		4.60	4.80	5.09	5.88	6.41	9.23
		10.48	11.14	14.91	15.41	20.80	23.56
		26.84	31.98	41.35	47.38		

The estimates of *θ* are calculated for the complete case and the three censoring scheme with effective sample sizes m_1_ = 26, m_2_ = 22. Results are summarized in Table 19. I is notable that *θ* is less than 0.5 for both estimates, which means that data 2 has a higher probability of having longer survival times than data 1. Moreover, estimates of *θ* based on the adaptive type-II hybrid progressive samples are close to those of complete data.

**Table 19:** Estimates of *θ* for example 2.

C.s	Complete	1	2	3
MLE	0.3444	0.2925	0.3482	0.3131
MPSE	0.3439	0.2907	0.3475	0.3142

Next, 95% Bootstrap confidence intervals are computed for all calculated estimators of *θ*. As shown in Table 20, it is clear that all estimates of *θ* lie inside the bootstrap confidence intervals.

**Table 20:** Bootstrap confidence intervals for each estimate of *θ* for example 2.

C.s	1	2	3
MLE	(0.1287, 0.548)	(0.2243, 0.5102)	(0.1607, 0.5563)
MPSE	(0.1276, 0.5495)	(0.2243, 0.5127)	(0.1618, 0.5592)

Furthermore, we calculate the standard error and average values resulted from bootstrapping for each estimate. From Table 21, we note that the standard error is the least for all the estimates of *θ* under the second censoring scheme. Average values of the estimates of *θ* are close to those in Table 19.

**Table 21:** Standard error and average value for each estimate after bootstrapping for example 2.

Standard error				Average value			
C.s	1	2	3	C.s	1	2	3
MLE	0.1175	0.07964	0.1106	MLE	0.3149	0.3674	0.3391
MPSE	0.1191	0.08003	0.1111	MPSE	0.3142	0.3669	0.3402

## Conclusions and recommendations

In life testing and reliability studies, progressive censoring is widely used to resolve many concerns that face experimenters for different types of experiments, such as reducing total test time, conserving experimental units, and estimating efficiently. However, there is always a trade-off between these three concerns to reduce the cost and the total test time of the experiment. Different types of progressive censoring have been developed to help reduce these concerns. The adaptive type-II progressive hybrid censoring allows more flexibility during the experiment and provides more control over the experiment hence, resulting in a shorter test duration and more failures to observe.

In article, we study the statistical inference of the SSR model under adaptive type-II progressive hybrid censoring when the random stress and strength components are IWD random variables that share the same shape parameter. We compared the performance of the MLE and the MPSE. It has been discovered that the MPSE has a smaller Bias and MSE for large and small sample sizes. Hence, we recommended using MPSE for estimating the reliability under adaptive type-II progressive hybrid censoring of the IWD under the second censoring scheme, where the random variables are independent and have common shape parameters.
